# Construction and validation of nomogram model for chronic postsurgical pain in patients after total knee arthroplasty: A retrospective study

**DOI:** 10.12669/pjms.41.3.11525

**Published:** 2025-03

**Authors:** Shenghao Zhao, Ying Hu, Ye Li, Jie Tang

**Affiliations:** 1 Shenghao Zhao Department of Bone and Joint Surgery, Wuhan Fourth Hospital, 76 Jiefang Ave, Wuhan, Hubei Province 430034, P.R. China; 2 Ying Hu Department of Ophthalmology, Renmin Hospital of Wuhan University, 99 Zhangzhidong Road, Wuhan, Hubei Province 430060, P.R. China; 3 Ye Li Department of Bone and Joint Surgery, Wuhan Fourth Hospital, 76 Jiefang Ave, Wuhan, Hubei Province 430034, P.R. China; 4 Jie Tang Department of Bone and Joint Surgery, Wuhan Fourth Hospital, 76 Jiefang Ave, Wuhan, Hubei Province 430034, P.R. China

**Keywords:** Chronic postsurgical pain, Nomogram model, Orthopedics, Risk factors, Total knee arthroplasty

## Abstract

**Objective:**

Chronic postsurgical pain (CPSP) after total knee arthroplasty (TKA) is the most common postoperative complication in orthopedics. This study aims to explore the risk factors for CPSP after TKA and construct a nomogram model.

**Methods:**

This retrospective study included clinical records of 430 patients who received TKA treatment at Wuhan Fourth Hospital between January 2020 to January 2024. Patients were randomly divided into a training cohort (n=301) and a validation cohort (n=129) in a 7:3 ratios. The Least Absolute Shrinkage and Selection Operator (LASSO) algorithm and logistic regression analysis were used to identify the independent risk factors, and a predictive nomogram model was established based on the identified risk factors. The concordance index (C-index), calibration curve, receiver operating characteristic (ROC) curve and decision curve analysis were used to assess the predictive accuracy and clinical application value of the nomogram model.

**Results:**

Six risk factors for predicting CPSP were identified, including preoperative anxiety, preoperative depression, preoperative pain, duration of tourniquet use, pain upon discharge, and postoperative C-reactive protein levels. The nomogram model demonstrated sufficient predictive accuracy, with the area under the curve (AUC) values of 0.761 (95% CI: 0.689-0.833) and 0.806 (95% CI: 0.700-0.911) in the training cohort and validation cohort, respectively. The C-index of the training cohort and validation cohort were 0.733 and 0.761, respectively. The calibration curve shows good consistency between the predicted risk of the model and the actual risk of CPSP. Decision curve analysis (DCA) demonstrated the clinical applicability of the model.

**Conclusions:**

The nomogram model established in this study for predicting CPSP after TKA has good predictive value and may be used in clinical practice to identify patients at high risk of developing CPSP after TKA.

## INTRODUCTION

Total knee arthroplasty (TKA) is a commonly used surgical procedure for treating knee osteoarthritis, medial and lateral knee deformities, rheumatoid arthritis, traumatic knee arthritis, and other diseases.^1^–^3^ TKA can improve knee joint function in patients by replacing damaged knee joints.^1^,^2^ Previous studies have shown that 10% to 34% of TKA patients develop chronic postsurgical pain (CPSP) 3-6 months after the surgery.^4^,^5^ CPSP, which may last from several months to several years, not only affects the rehabilitation effect of knee joint function and increases the economic burden on patients but also seriously affects their quality of life.^3^–^5^

Nevertheless, current clinical research focuses on acute postoperative pain after TKA surgery, with little attention paid to CPSP, and no definite and effective CPSP management plan.^5^ Although some studies have analyzed the risk factors for CPSP in TKA patients, a unified risk prediction model has not yet been established.^5^,^6^ This study aimed to develop a practical and reliable prediction model for the occurrence of CPSP by combining common clinical variables. Our results may help clinicians identify TKA patients at high risk of developing CPSP in a timely manner.

## METHODS

**Fig.1:**
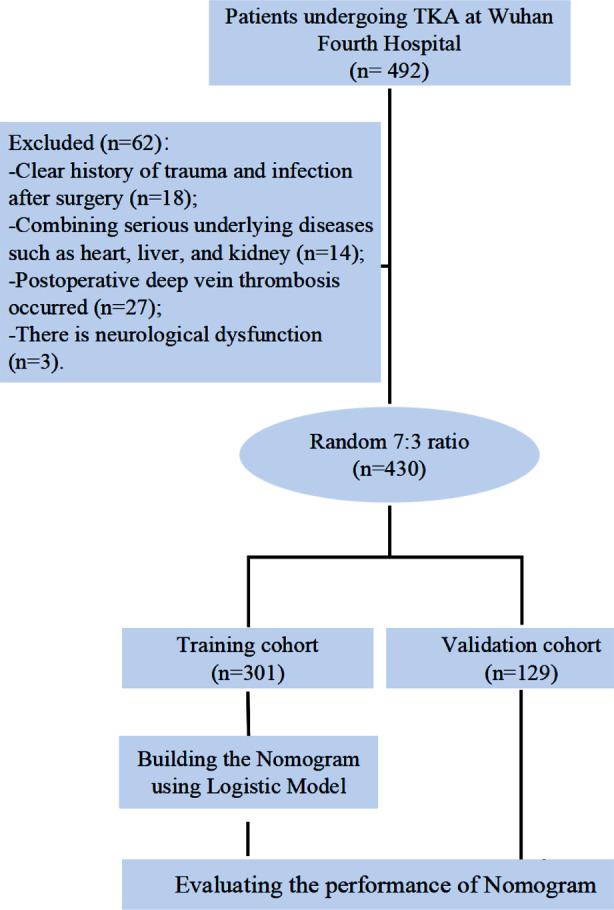
Participant screening process diagram.

This retrospective study included clinical records of all patients who received TKA treatment at Wuhan Fourth Hospital between January 2020 to January 2024 were screened for eligibility. The screening process is shown in Fig.1.

### Ethical Approval:

Our hospital’s ethics committee approved the study, with the number KY2024-148-01 and the date 2024 July 19^th^.

### Inclusion criteria:

- Over 18 years old.
- Met the diagnostic criteria for knee osteoarthritis.^7^
- First-time TKA treatment, completed by the same surgical team.
- Follow-up of at least six months.

### Exclusion criteria:

- Clear history of trauma or infection after the surgery.
- Serious comorbidities such as heart, liver, and kidney diseases.
- Postoperative deep vein thrombosis.
- Neurological dysfunction.
- Incomplete clinical records.

### Data collection:

General information, including gender, age, BMI, education level, and sleep quality. Disease conditions, including disease duration, history of chronic diseases (hypertension, type-2 diabetes, coronary heart disease, and stroke.

Perioperative conditions, including preoperative anxiety and depression, unilateral or bilateral joint replacement, preoperative pain level, preoperative knee joint function, duration of tourniquet use, intraoperative bleeding, length of hospital stay, degree of pain at discharge, calcium use, use of painkillers, and C-reactive protein levels one day after the surgery.

### Sleep quality assessment:

Sleep quality assessment was done using the Pittsburgh Sleep Quality Index scale, with a total score range of 0-21 points. A score of 0-5 indicates good sleep quality; a score of 6-10 indicates good sleep quality; a score of 11-15 indicates average sleep quality; a score of 16-21 indicates poor sleep quality.^8^

### Preoperative anxiety and depression assessment methods:

The Hospital Anxiety and Depression Scale (HADS) was one day before surgery to measure the patient’s anxiety and depression levels. HADS includes two parts: anxiety (A) and depression self-assessment form (D), with a total score of 0-21 points. A score of ≥ 9 indicates the presence of anxiety or inhibition in the patient.^9^

### Preoperative knee joint function assessment:

Using the Hospital for Special Surgery (HSS) scoring system, the patient’s pain, functional activity, knee joint range of motion, knee flexion deformity, muscle strength, and knee joint stability were observed. According to the total score, the knee joint function was considered excellent with a score of ≥ 85, good with a score of 70-84, medium with a score of 60-69, and poor with a score of ≤ 59.^10^

### Definition of postoperative CPSP:

- New pain at the surgical site or adjacent areas of the knee joint after the surgery;
- Pain caused by other factors (such as chronic infection or advanced cancer pain) was excluded;
- In cases of preexisting pain at the surgical site (before the surgery), CPSP after TKA was diagnosed if the nature of the pain changed after TKA.^4^

Patients were followed up for six months after the TKA surgery. The degree of pain was evaluated using the Visual Analog Scale (VAS). VAS uses a 10cm straight line, with 0 indicating complete lack of pain and 10 indicating unbearable pain. The patient draws the position on the VAS paper based on the degree of pain; 1-3 points indicate mild pain, 4-6 points indicate moderate pain, and ≥ 7 points indicate severe pain.^11^ Mild pain is defined as pain that does not affect daily activities and sleep and does not require medication.

### Statistical analysis:

Data analysis was done using SPSS version 26.0 (IBM Corp, Armonk, NY, USA). The normality of the distribution of continuous variables was evaluated using the Shapiro-Wilk test. Non-normally distributed data were represented as the median and interquartile range (IQR), and the Mann-Whitney U test was used to determine inter-group differences. The count data were represented by [example (%)], and the chi-square test was used for comparison between groups. The Least Absolute Shrinkage and Selection Operator (Lasso) regression was used to screen for independent prognostic factors.

The predictor variables selected by Lasso regression were merged into logistic regression to construct a nomogram. Based on the predictive model, the performance of the nomogram model was evaluated in both the training and the validation cohort. The receiver operating characteristic (ROC) and calibration curves were used to evaluate the model’s predictive performance. Briefly, the area under the curve (AUC) of the ROC curve may range from 0.50 to 1.00, and AUC closer to one indicates better prediction performance. The calibration process checks whether the predicted risks are consistent with the observed risks. A decision curve analysis (DCA) was used to evaluate the clinical practicality of the model and assess its effectiveness. P<0.05 indicated statistical significance.

## RESULTS

**Fig.2:**
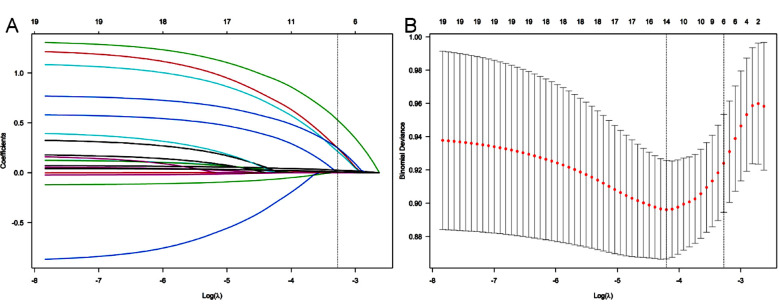
LASSO coefficient curve of CPSP in TKA patients. A: Each curve in the graph represents the coefficient variation of each variable; The vertical axis represents the coefficient values, the lower horizontal axis represents log (λ), and the upper horizontal axis represents the number of non-zero coefficients in the model at each time point. B: 10-fold cross-validation fitting.

A total of 430 TKA patients were included in this study and randomly divided into two cohorts in a 7:3 ratio. The training cohort comprised 301 patients, while 129 patients were assigned to the validation cohort. The clinical characteristics of the patients are shown in Table-I. During the follow-up period, the incidence of CPSP in the training and validation cohorts was 18.27% (55/301) and 19.38% (25/129), respectively, with no statistically significant difference between the two cohorts (*P*=0.520). In the training cohort, we employed the LASSO regression algorithm to perform feature selection. This approach helps minimize the impact of multicollinearity and offers strong predictability and stability. We selected features based on the partial likelihood binomial deviance reaching its minimum value, and six variables with nonzero coefficients were retained in the LASSO regression (Fig.2). These variables were considered significantly associated with CPSP. The identified variables included including preoperative anxiety, preoperative depression, preoperative pain VAS, duration of use of tourniquet, pain VAS score at discharge, and C-reactive protein (CRP) one day after surgery level.

**Table-I:** Clinical Characteristics of Patients.

Characteristics	Training cohort (n=301)	Validation cohort (n=129)	Z/χ²	P
Male (yes),n(%)	177 (58.8)	80 (62.0)	0.387	0.534
Age (years), M(P25/P75)	62 (54-69)	65 (58-70)	-1.208	0.227
BMI (kg/m²), M(P25/P75)	23.5 (21.4-26.2)	23.9 (21.5-26.5)	-0.757	0.449
Educational level, n(%)			0.1	0.752
Junior high school and below	203 (67.4)	98 (32.6)		
High school and above	89 (69.0)	40 (31.0)		
Sleep			1.148	0.765
Good sleep quality (0-5)	79 (26.2)	37 (28.7)		
The sleep quality is decent (6-10)	133 (44.2)	59 (45.7)		
Sleep quality is average (11-15)	65 (21.6)	26 (20.2)		
Poor sleep quality (16-21)	24 (8.0)	7 (5.4)		
Duration of illness (years), M(P25/P75)	5 (4-6)	5 (3-6)	-0.379	0.705
Combined chronic diseases, n(%)			2.368	0.500
Nothing	79 (26.2)	39 (30.2)		
1 type	123 (40.9)	57 (44.2)		
2 types	92 (30.6)	31 (24.0)		
3 types or above	7 (2.3)	2 (1.6)		
Preoperative anxiety (yes),n(%)	81 (26.9)	33 (25.6)	0.082	0.775
Preoperative depression (yes), n(%)	70 (23.3)	35 (27.1)	0.735	0.391
Unilateral TKA (Yes), n(%)	265 (88.0)	116 (89.9)	0.317	0.573
Preoperative pain VAS score, n(%)			0.579	0.447
≤6	205 (68.1)	96 (31.9)		
≥7	83 (64.3)	46 (35.7)		
Preoperative HSS score (points), M(P25/P75)	58 (52-61)	56 (50-62)	-1.343	0.179
Duration of use of tourniquet (minute), M(P25/P75)	59 (55-64)	60 (53-68)	-0.269	0.788
Intraoperative bleeding volume (mL), M(P25/P75)	47 (36-56)	42 (35-54)	-1.677	0.094
Length of hospital stay (days), M(P25/P75)	10 (9-11)	9 (8-11)	-1.074	0.283
Pain VAS score at discharge, n(%)			0.038	0.845
≤3	226 (75.1)	75 (24.9)		
≥4	99 (76.0)	31 (24.0)		
Taking calcium supplements (yes), n(%)	209 (69.4)	76 (0.034)	4.472	0.034
The use of painkillers (Yes), n(%)	233 (77.4)	106 (82.2)	1.227	0.268
CRP level one day after surgery (mg/L), (P25/P75)	38 (32-47)	36 (34-51)	-0.106	0.916
CPSP (yes), n(%)	55 (18.27)	27 (20.93)	0.413	0.520

BMI: body mass index; VAS: visual analog scale; TKA: total knee arthroplasty; HSS: Hospital for Special Surgery scoring system; CRP: C-reactive protein; CPSP: chronic postsurgical pain.

To further investigate their predictive significance, we conducted a multivariate logistic regression analysis using these six variables. The results demonstrated that preoperative anxiety [odds ratio (OR) = 2.689; 95% confidence interval (CI) = 1.355-5.338; *P* = 0.005], preoperative depression (OR = 3.453; 95%CI = 1.733-6.882; *P* < 0.001), preoperative pain VAS score (left side) (OR = 2.368; 95%CI = 1.218-4.608; *P* = 0.011), duration of use of tourniquet (OR = 1.060; 95%CI = 1.018-1.105; *P* = 0.005), pain VAS score at discharge (OR = 2.310; 95%CI = 1.165-4.580; *P* = 0.017), and CRP one day after surgery (OR = 1.306; 95%CI = 1.003-1.070; *P* = 0.031) were all significant predictors of CPSP. The detailed results of the multivariate logistic regression analysis can be found in Table-II.

**Table-II:** Multivariate logistic regression analysis of predictive factors selected through LASSO regression.

Independent variables	B	95% CI	P
Preoperative anxiety	0.989	2.689(1.355~5.338)	0.005
Preoperative depression	1.239	3.453(1.733~6.882)	<0.001
Preoperative pain VAS score	0.862	2.368(1.218~4.608)	0.011
Duration of use of tourniquet	0.059	1.060(1.018~1.105)	0.005
Pain VAS score at discharge	0.837	2.310(1.165~4.580)	0.017
CRP one day after surgery	0.035	1.036(1.003~1.070)	0.031

***Note:*** VAS: visual analog scale; CRP: C-reactive protein; B is the regression coefficient.

**Fig.3:**
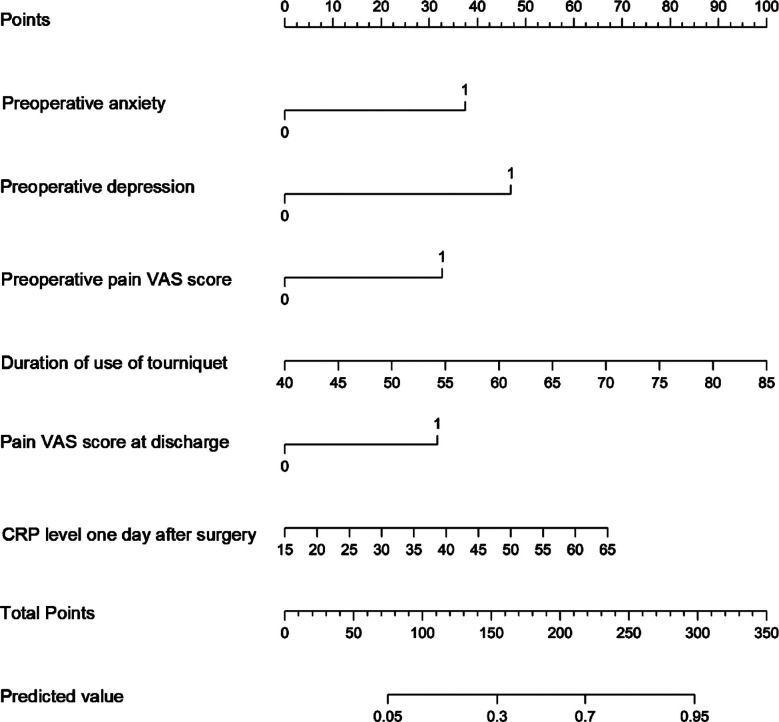
Nomogram of CPSP after TKA. Each level of the predictor variable represents a specific score. The total score is generated by summarizing the scores of each predictor variable. The total score corresponds to the CPSP probability.

**Fig.4:**
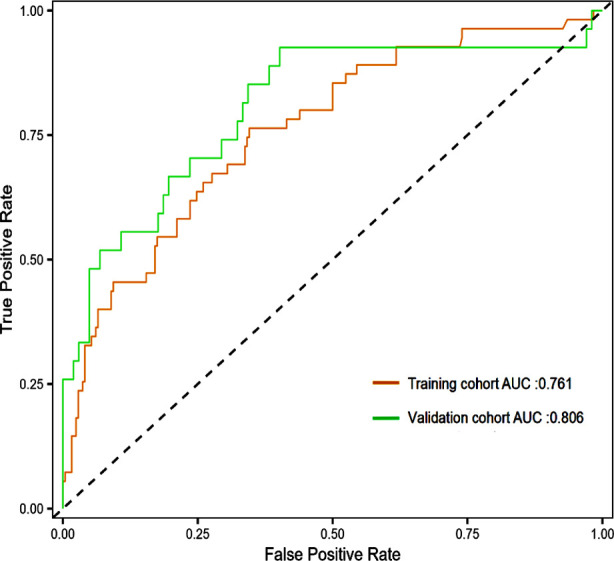
ROC curve and AUC of the predictive model; ROC: receiver operating characteristic; AUC: area under the curve.

**Fig.5:**
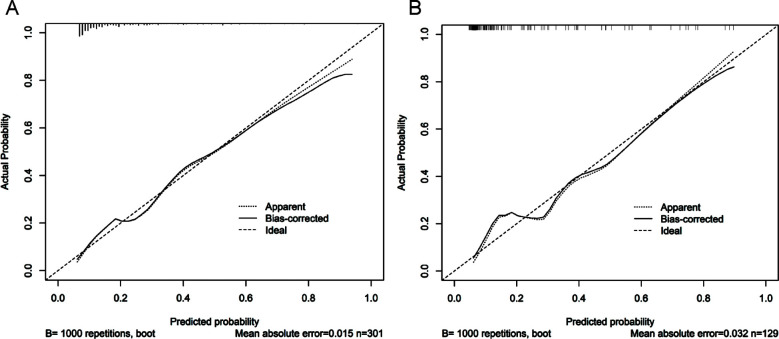
Calibration diagram of the prediction model. A. Calibration chart of the training cohort. B. Calibration chart in the internal validation cohort. The x-axis represents the predicted probability of CPSP. The y-axis represents the observed CPSP. The diagonal dashed line represents the perfect prediction of the ideal model. The solid line represents the performance of the nomogram. It indicates that solid lines are closer to diagonal dashed lines for better prediction, indicating that the model has good predictive ability.

**Fig.6:**
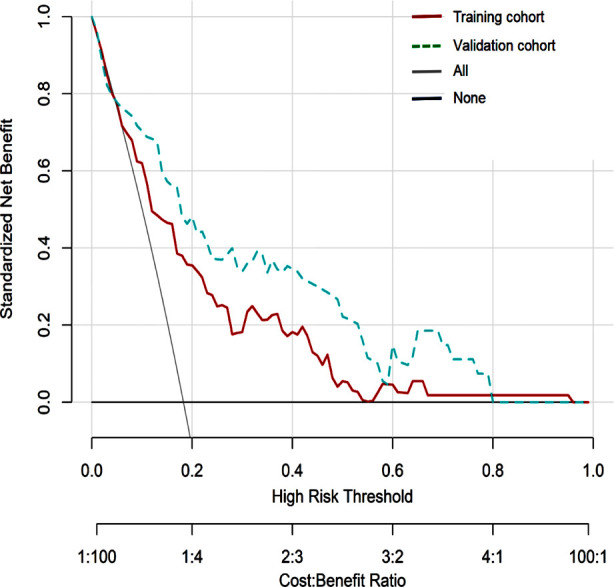
DCA of the nomogram. DCA: decision curve analysis.

A nomogram model for predicting the risk of CPSP occurrence after TKA was constructed based on the identified six independent risk factors (Fig.3). According to the nomogram, the sum of the score values corresponding to each predictive indicator was recorded as the total score. The predicted probability corresponding to the total score indicated the risk of CPSP after TKA. The Hosmer Lemeshow test showed that the training cohort had a chi-square of 5.763, *P*=0.674, and the internal validation cohort had a chi-square of 9.880, *P*=0.196, indicating that the predicted results were close to the observed results. The ROC curve in the training cohort showed good discriminability (AUC: 0.761; 95% CI: 0.689-0.833). The discriminative performance of the model was further validated in the validation cohort (0.806; 0.700-0.911) (Fig.4). The C-index of the training cohort and validation cohort were 0.733 and 0.761, respectively. In addition, calibration curve analysis shows good consistency between the predicted probability and the observed occurrence of CPSP in both cohorts (Fig.5). DCA further demonstrated the clinical applicability of the model (Fig.6).

## DISCUSSION

This retrospective study identified preoperative anxiety, preoperative depression, preoperative pain, duration of tourniquet use, pain upon discharge, and postoperative C-reactive protein levels as independent risk factors of CPSP after TKA. The constructed nomogram model demonstrated a good predictive value. The incidence of CPSP after TKA in our study was 19.07% (82/430). Our results align with the study of Tang S et al.,^12^ which showed that the incidence of CPSP after TKA was 20.9%. Similarly, a systematic review by Wylde et al.^13^ demonstrated that the incidence of CPSP after TKA is about 20%, which is consistent with our results. Our results further confirm that with the gradual aging of the population and the increasing number of patients undergoing TKA, the occurrence of CPSP after TAK still remains a pressing concern. Therefore, identifying risk factors of CPSP is crucial for timely diagnosis and intervention.

Our analysis identified preoperative anxiety and depression, preoperative pain, duration of tourniquet use, pain upon discharge, and one-day postoperative CRP levels as independent risk factors for CPSP in TKA patients. A nomogram model constructed using these independent risk factors and validated with internal and external data demonstrated good discriminative power and calibration. Qian J et al.^14^ also constructed a predictive model for CPSP in TKA patients that incorporated gender, postoperative 24-hours pain level, and HSS score three months after the surgery. In this study, we show that in addition to demographic characteristics and pain levels, psychological factors and CRP are also independent risk factors for CPSP in TKA patients. Moreover, this study used external data for validation and showed that the model’s predictive ability is reliable. A review by Kim DH et al.^15^ also stated that perioperative pain, physical function, psychological state, surgical factors, etc., are potential factors for CPSP after TKA, which is consistent with the results of this study.

Patients undergoing TKA surgery experience long-term pain before the surgery and often have psychological problems such as anxiety and depression. A meta-analysis of Springborg AH et al.^16^ included 18 studies and showed that mental health is a predictor of pain after TKA. Fernández de Las Pe ñ as C et al.^17^ also indicated that preoperative pain and mental health are highly recognized predictive factors for CPSP after TKA. Sydora BC et al.^18^ found that preoperative pain, depression, anxiety, and severe pain were risk factors for CPSP after TKA. Together with these results, our study further emphasizes the importance of assessing the preoperative psychological state of patients undergoing TKA. Targeted psychological interventions are needed for patients with anxiety, depression, and catastrophic pain to prevent the development of post-TKA CPSP.

Tourniquet is widely used in knee joint replacement surgery to reduce intraoperative bleeding and shorten the time of the procedure.^19^ However, there is still some controversy regarding the use of tourniquets. Wang et al.^20^ found that the use of a tourniquet during TKA was not associated with a significant difference in knee joint function, incidence of complications, and satisfaction. In contrast, Wang K et al.^21^ found that in the early recovery period after TKA, short-term use of tourniquets may lead to faster recovery and less pain. This study showed that CPSP was associated with significantly longer use of tourniquets, and their use was identified as an independent risk factor for CPSP. It is plausible that prolonged tourniquet use may cause long-term ischemia-reperfusion injury that may lead to pain response and increased risk of muscle injury and postoperative pain.^22^ Therefore, tourniquets after TKA should be strictly controlled to reduce the probability of CPSP.

The results of this study showed that the intensity of pain at discharge and high CRP levels one day after the surgery were also independent influencing factors for CPSP after TKA. Knezevic NN et al.^23^ found a significant correlation between early postoperative severe pain and the incidence of CPSP after TKA, which is consistent with our results. Therefore, for patients undergoing TKA, effective pain management should be given in the early postoperative period to avoid acute and severe pain, thereby reducing the risk of CPSP after TKA. Postoperative chronic knee joint swelling in TKA patients may be related to local inflammation, edema, or accumulation of blood and fluid. The observed increase in postoperative CRP indicates persistent chronic inflammation, which increases the sensitivity of the peripheral and central nervous systems,^24^ leading to the development of CPSP.

This study generated a predictive nomogram model for CPSP after TKA that incorporated independent risk factors of CPSP and demonstrated a good predictive value. Independent risk factors identified in our study are easy to measure, are readily available, and were established in a well-characterized training cohort of patients undergoing TKA. Our model can be used in clinical practice for personalized assessment of patients. Scores for various influencing factors may be used to calculate the total score through a nomogram. Our nomogram model has demonstrated good discriminability, with C-indexes of 0.733 and 0.761 for the training and validation cohorts, respectively. Health practitioners may use this easy-to-use model to assess the risk of developing CPSP after TKA and identify high-risk populations as early as possible.

### Limitations:

This is a retrospective study with a small number of patients who did not experience CPSP, which increases the risk of selection bias. Additionally, our study did not analyze metabolic factors and inflammatory indicators that may be related to CPSP, such as white blood cell count and procalcitonin. Including these indicators may help improve the predictive accuracy of the model. Furthermore, this is a single-center study. To validate the prediction model, we randomly divided the entire cohort into a training and an internal validation cohort in a 7:3 ratios. However, the model was not validated by an external validation cohort. Future prospective multicenter studies with larger sample sizes are needed to further confirm the predictive performance of our predictive model. Finally, models based on more advanced machine learning algorithms or radiomics may be more helpful in providing predictive accuracy.

## CONCLUSION

In summary, our study suggests that preoperative anxiety and depression, preoperative pain, duration of tourniquet use, pain upon discharge, and postoperative C-reactive protein levels are predictive indicators of CPSP after TKA. We have constructed a nomogram model with excellent discrimination and accurate calibration to predict the risk of CPSP occurrence. Our nomogram allows the use of routine clinical data to calculate CPSP recurrence probability and to identify patients at high risk of developing CPSP after TKA. The results of this study may contribute to developing personalized treatment and management plans to reduce the incidence of CPSP.

### Authors’ contributions:

**SZ and YH:** Study design, literature search and manuscript writing.

**YL and JT:** Data collection, data analysis and interpretation. Critical Review

**SZ and YH:** Were involved in the manuscript revision and validation.

All authors have read and approved the final manuscript and are accountable for the integrity of the study.
